# Effects of the removal or reduction in density of the malaria mosquito, *Anopheles gambiae s.l*., on interacting predators and competitors in local ecosystems

**DOI:** 10.1111/mve.12327

**Published:** 2018-07-25

**Authors:** C. M. Collins, J. A. S. Bonds, M. M. Quinlan, J. D. Mumford

**Affiliations:** ^1^ Centre for Environmental Policy Imperial College London London U.K.; ^2^ Bonds Consulting Group LLC Panama City Beach, Florida U.S.A.

**Keywords:** Competition, ecology, environmental impact assessment, environmental risk assessment, mosquito, malaria, predation, vector control

## Abstract

New genetic control methods for mosquitoes may reduce vector species without direct effects on other species or the physical environment common with insecticides or drainage. Effects on predators and competitors could, however, be a concern as *Anopheles gambiae s.l*. is preyed upon in all life stages. We overview the literature and assess the strength of the ecological interactions identified. Most predators identified consume many other insect species and there is no evidence that any species preys exclusively on any anopheline mosquito. There is one predatory species with a specialisation on blood‐fed mosquitoes including *An. gambiae s.l*.. Evarcha culicivora is a jumping spider, known as the vampire spider, found around Lake Victoria. There is no evidence that these salticids require *Anopheles* mosquitoes and will readily consume blood‐fed *Culex*. Interspecific competition studies focus on other mosquitoes of larval habitats. Many of these take place in artificial cosms and give contrasting results to semi‐field studies. This may limit their extrapolation regarding the potential impact of reduced *An. gambiae* numbers. Previous mosquito control interventions are informative and identify competitive release and niche opportunism; so while the identity and relative abundance of the species present may change, the biomass available to predators may not.

## Introduction

Malaria remains one of the most prevalent and deadly diseases in the tropical regions. In 2016 there were 216 million cases of malaria globally (95% confidence interval: 196–263 million) and 450 000 malaria deaths, 90% of which occurred in sub‐Saharan Africa [World Health Organization (WHO) 2017]. Members of the *Anopheles gambiae* complex, which are widely distributed throughout sub‐Saharan Africa, are responsible for much of the transmission of malaria across this largely endemic area (Coetzee *et al*., 2000; Sinka *et al*., 2012). The *An. gambiae* complex or *An. gambiae sensu lato* (*s.l*.) was recognized as a species complex in the 1960s and currently includes eight acknowledged species: *Anopheles amharicus*, *Anopheles arabiensis*, *Anopheles bwambae*, *Anopheles coluzzii (*formerly *An. gambiae* M form*)*, *An. gambiae (*formerly *An. gambiae* S form*)*, *Anopheles melas*, *Anopheles merus* and *Anopheles quadriannulatus* (Sinka *et al*., 2010; Coetzee *et al*., 2013). Before *An. coluzzii* and *An. gambiae* were recognized as separate species, they were jointly referred to as *An. gambiae sensu stricto* (*s.s*.) and essentially all of the literature prior to 2010 and many publications since refer to both species under this name. More typically, much of the literature refers to *An. gambiae* without distinguishing between *s.l*. and *s.s*.; the present work will follow the nomenclature of the source literature. Although the ability to transmit malaria parasites to humans varies greatly among the different species of the complex, *An. gambiae*, *An. coluzzii* and *An. arabiensis* are considered the primary malaria vectors in Africa.

Vector control, including habitat alteration, has been the primary focus of malaria eradication and control attempts. The two major strategies for vector control are the use of insecticide‐treated bednets (ITNs) and indoor residual spraying (IRS). These methods have been successful in suppressing the transmission of malaria but are not sufficient in areas of sub‐Saharan Africa in which the entomological inoculation rate (EIR) exceeds 1000 infectious bites/person/year (Ferguson *et al*., 2010). A major challenge to these vector control approaches is fast‐spreading insecticide resistance (Abilio *et al*., 2011; Chanda *et al*., 2011; Ranson *et al*., 2011; Hemingway *et al*., 2016).

The incomplete and increasingly precarious success of traditional malaria control methods brings a growing demand for novel and improved techniques (WHO, 2008). Since the introduction of the sterile insect technique (SIT), modern molecular biology has led to genetic strategies that have attracted increasing interest as complements to established methods (Kitzmiller, 1972; Windbichler *et al*., 2007; Alphey, 2014; McLean & Jacobs‐Lorena, 2016). Genetic approaches can suppress mosquito populations or enable infiltration to wild mosquito populations of genetic modifications that render them refractory to malaria parasite infection. These innovative genetic methods, and vector control methods generally, need to gain responsible, informed public understanding and regulatory appraisal before approval for field testing can be sought (Emerson *et al*., 2017). They offer unique opportunities to eliminate or significantly reduce the density of the target vector species. This focuses environmental risk assessment attention on the indirect effects of such specific targeted control on non‐target organisms associated with the vector [European Food Safety Authority (EFSA), 1989]. Such assessments would specifically address the potential loss of beneficial predatory species and increases in harmful competing species.

Synthesizing current knowledge of the ecology of the African Anophelines contributes to evaluating any potential ecological implications of a population reduction or removal. Mosquitoes interact with many different organisms, which may affect their dynamics: they are eaten, parasitized and infected by natural enemies, and they compete for resources with other animals and, in particular, with other mosquito species (Godfray, 2013).

Adult anopheline mosquitoes, as flying insects, form a small part of the insect biomass on which insectivores across Africa feed. *Anopheles gambiae* is a relatively small mosquito with a mean ± standard deviation adult female dry mass of 0.25 ± 0.03 mg, which represents half to one‐third the mass of many *Aedes* (Diptera: Culicidae) and *Culex* mosquito species (Van Handel, 1965; Nasci, 1990; Koella & Lyimo, 1996). Males are typically smaller than females and, based on numbers estimated from sequential mark–release–recapture studies in a West African village, there is a potential biomass of 50–235 g/km^2^ in the wet season and roughly 10% of that in the dry (Epopa *et al*., 2017). Foraging theory predicts that these small, mobile insects of low profitability (energy gained over hunting and handling time) are, unless densely aggregated, unlikely to be an attractive food source that optimizes fitness and energy intake (Stephens *et al*., 2007). Although many different animals eat mosquitoes, it is generally recognized that the majority of these predators are polyphagous and consume mosquitoes only in addition to other small aerial invertebrates (Findley & Black, 1983).

The aquatic larval and pupal life stages are also fed upon by many invertebrate species and by larvivorous fish (Service, 1977; Ohba *et al*., 2010; Dida *et al*., 2015). Of interest here is the role of other mosquito species as competitors of *An. gambiae*. Two critical questions concern, respectively, whether population reduction might permit an increase in the density of another species, and whether the ecological niche previously occupied by *An. gambiae* might be assumed by another of the mosquito species able to transmit malaria (McCann *et al*., 2014).

## Materials and methods

The present work reviews the literature available on ecological interactions of the principal malaria vectors in the *An. gambiae* complex with their predators and competitors in natural habitats in order to summarize current knowledge. All papers cited were reviewed, but not all of the studies that were built on in these investigations are included. The present authors do not claim to have been exhaustive and the presentation of all studies is beyond the scope of this paper. Where relevant, such as with birds and bats, the predation of mosquitoes more widely is included. The aim of this paper is to provide an overview of the organisms already considered in the literature and to contribute to ongoing discussions on the potential ecological implications of any malaria vector control initiative that seeks to reduce populations of a single species of the *An. gambiae* complex at an area‐wide scale.

Peer‐reviewed scientific literature, relevant internal reports and web‐based resources were searched to identify *An. gambiae s.l*. predators and competitors. Topic search terms, used individually and in combination, included ‘*Anopheles*’, ‘*Anopheles gambiae*’, ‘Africa’, ‘complex’, ‘vector’, ‘malaria’, ‘prey’, ‘predator’, ‘competitor’ and ‘control’. The Web of Science, Google Scholar, Google and Mendeley databases were all used to explore the field, as were citations within identified papers. Searches took place during March–June 2017.

The literature is diverse. It reflects the spectrum of study approaches that range from very controlled microcosm laboratory experiments to extended time and space field observations. Small‐scale laboratory studies can provide a baseline from which to identify and characterize further ecological linkages. Extrapolations or inferences on the strength and stability of an ecological linkage from these laboratory studies are presented with caution. A feeding preference evaluated in a simple choice setting cannot be concluded to provide evidence of a dependence or even a requirement as part of a natural diet. The competitive environment and what a predator eats in the field are variable and reflect environmental conditions, as well as inherent decision making by individual animals.

## Results

### Anopheles gambiae s.l. mosquitoes as a resource

#### Lifecycle

As with all dipteran species, anopheline mosquitoes have a fully metamorphic (holometabolous) lifecycle. Winged adults mate, after which the female seeks a bloodmeal from a vertebrate host [generally human (Garrett‐Jones *et al*., 1980; Githeko *et al*., 1994)] with which to mature and develop her eggs. Females oviposit in usually shallow and often temporary water bodies and the eggs hatch into the larval stage. Development from egg to adult via aquatic larval and pupal stages is temperature‐ and environment‐dependent and takes 10–25 days (Bayoh & Lindsay, 2003). After pupation, the adult emerges to mature and feed on nectar before mating (Service & Towson, 2002). The lifespan of the adult *Anopheles* mosquito is a few weeks for males and typically less than a month for females, although recent evidence of aestivation in *An. coluzzii* suggests this may be substantially longer during a dry season (Lehmann *et al*., 2010; Dao *et al*., 2014).

#### Dispersion in space and time

During the larval and pupal stages, biomass is restricted to the water bodies in which the eggs hatched and, at this time and especially in small water bodies, can be concentrated at high densities. High density and site‐specific factors contribute to low survival overall and high mortality in all developmental stages (Koenraadt *et al*., 2004aa; Munga *et al*., 2007).

Maturation in both sexes, and feeding and resting in males, are largely disaggregated activities that reduce the predictability of adults as a resource to predators. Additionally, adults are harder to locate and catch relative to larvae and are of low resource value compared with other flying prey such as Lepidoptera (butterflies and moths) (Gonsalves *et al*., 2013). Much of 20th century vector control success with ITNs and IRS was based on post‐mating aggregation in females as they sought to feed on sleeping humans and then to rest in houses. Another period in the adult stage during which anopheline mosquitoes are concentrated as a resource occurs during swarming activity at twilight. Males aggregate above specific, visually contrasting markers and females enter these swarms to find a mate (Diabaté *et al*., 2011). A large swarm of 1000 mosquitoes can contain approximately 0.2 g of mosquito biomass, and in such concentrated groups some predation of the adult stage by dragonflies is observed (Yuval & Bouskila, 1993).

The biomass of *An. gambiae s.s*. mosquitoes present in space and time has two strong correlates: the availability of standing water for larval development, and host availability for bloodmeals and egg maturation. The first drives a strongly seasonal pattern of abundance across much of the sub‐Saharan range and the second influences the spatial dispersion of mosquitoes in and around human habitations and villages (Sinka *et al*., 2012; Yaro *et al*., 2012).

### Predators

Ecologically, predators may be described as ‘specialists’ (monophagic/stenophagic) or ‘generalists’ (polyphagic/euryphagic) based on whether their natural diets are ‘narrow’ or ‘broad’. Although such studies are rare, to understand the degree of association between any predator and either or both larval and adult *An. gambiae*, any investigation should seek to reflect the broader diet of the predator. The studies identified and included here, are, in the majority, observations and literature reviews that help to provide a balanced description of potential predatory paradigms. There are also some ‘no choice’ laboratory tests that investigate preference in terms of whether predators will or will not eat various life stages of *An. gambiae*. There are semi‐field tests that attempt to overlay some aspects of a natural habitat, but these experiments offer either no choice or only one other option. A few more complex field and laboratory trials have attempted to observe, under controlled conditions, the behaviour of predators with a variety of prey. There are also field captures of wild predators that identify the presence of *An. gambiae s.l*. in the gut. These are positive or negative responses with little information on volume ingested.

The available literature provides an overview of the published ecological relationships of *An. gambiae s.l*. The diet of mosquitoes as juveniles and adults has not been explored here.

Predation of larvaeThe natural enemies of mosquito larvae are many and diverse, and include insects, spiders, hydras, planaria, copepods, bats, birds and fish. Munga *et al*. (2007) identified seven families of mosquito predator in larval habitats, including Hydrophilidae (Coleoptera, water scavenger beetles), Dytiscidae (Coleoptera, diving beetles), Corixidae (Hemiptera, water boatmen), Nepidae (Hemiptera, water scorpions), Notonectidae (Hemiptera, backswimmers), Belostomatidae (Hemiptera, giant water bugs) and Cordulidae (Odonata, dragonflies). There are reports that combinations of predatory invertebrates can account for more than 90% of the natural mortality of immature stages of mosquitoes in some aquatic environments (Service, 1971, 1973, 1977).

Much of the literature on larval predation comes from the context of biological control of mosquitoes; many species have been proposed as possible contributors to this, but the oviposition choices of female *An. gambiae* affect predator encounter rates substantially. *Anopheles gambiae s.l*. larvae occur in a great variety of habitats, but the most important are small, shallow, sunlit and usually temporary pools (Minakawa *et al*., 2004). Because of the small size and transient nature of many of these water bodies, few predator species successfully colonize them and habitat stability is low in smaller habitats such as cattle hoof prints, ruts and swales. Environmental and bottom‐up effects, such as evaporation, flushing and reduced food sources in ephemeral opportunistic habitats may be stronger effects as the predation mortality of *An. gambiae* larvae in these habitats is likely to be relatively low. The most important invertebrate predators in temporary pools are likely to be turbellarian. Turbellarians (free‐living flatworms) assume an importance in ephemeral ponds because they can produce resting eggs that survive dry periods (Blaustein & Dumont, 1990). They are present and become active within the first few days of rains, whereas most other invertebrate predators become effective only later in the hydro‐period of individual pools or at later stages of the rainy season. The relative importance of predation and habitat effects are supported by higher larval survivorship (35–51%) in artificial, semi‐natural habitats not yet colonized by predators (Munga *et al*., 2006).

Marshes, rice fields, borrow‐pits and wells are examples of larger and more permanent larval habitats. These can support a variety of both invertebrate and vertebrate predators (Service, 1971, 1977). In rice fields, both predator densities and *Anopheles* spp. survivorship were found to vary greatly through the cropping season (13.4–84.5%); water depth, rice height and predation were primary contributors (Chandler & Highton, 1976).

By creating life tables, Service (1971, 1973) estimated overall larval mortality, from multiple sources including competition and predation, of 97.1% and 96.6% in stable ponds, which are similar to the mortality rates of 95.2% and 96.6% recorded previously in a marsh and pools. Samples of surface and aquatic larval predators found were tested using a precipitin reaction to identify ingestion of *An. gambiae*. Lycosid spiders (Arachnida, wolf spiders), Muscidae (Diptera, houseflies) and Coleopterans (beetles) were present in all habitats and large proportions tested positive. Of truly aquatic fauna, Odonata (dragonflies and damselflies) were not found in the more temporary habitats such as small pools and ditches, and, furthermore, did not test positive in any reactions (Service, 1971, 1973). Conversely, a later study found nine different species of Odonata, of which five responded positively for *An. gambiae* (Service, 1977). Similar *An. gambiae* overall larval mortality rates exceeding 93%, 95% and 98% in drainage ditches, cow hoof prints and disused goldmine habitats, respectively, have been estimated (Munga *et al*., 2007).

Polymerase chain reaction (PCR) analysis has also been used to determine whether mosquito predators in wetland habitats feed on *An. gambiae s.l*. larvae and, in one example, showed that, of 330 potential individual predators, 54.2% had ingested *An. gambiae*. The highest incidence of consumption was in Odonata (dragonfly larvae) (70.2%), followed by Hemiptera (water boatman bugs) (62.8%), Amphibia (tadpoles) (41.7%) and Coleoptera (beetles) (18.0%) (Ohba *et al*., 2010). Some of these tests can be influenced by the metabolic rate of the test subject. Schielke *et al*. (2007) used an optimized PCR technique with which intergenic spacer (IGS) ribosomal DNA (rDNA) of *An. gambiae s.l*. could be detected for longer after ingestion by Lestidae (Odonata, damselflies) after 4 h, by Libellulidae (Odonata, dragonflies) after 6 h, and by Notonectidae (Hemiptera, backswimmers) after 24 h.

The larvae of *An. gambiae s.l*. are thus consumed by a wide variety of predators. Literature available on this predation is presented by taxonomic order.

#### Flies (Diptera)

Several species of shore fly (Ephydridae) are aquatic predators and have been reported to eat Anophelines (Minakawa *et al*., 2007).

Experiments on prey choice in *Ochthera chalybescens* (Diptera: Ephydridae) suggest that prey larval stage and size do not affect predator capacity. Younger larvae spent more time near the water surface than did older larvae during experiments, and this behaviour increased the time for which they were exposed to predators. Older larvae can dive deeper, a behaviour that is considered a predator avoidance mechanism (Tuno *et al*., 2007). The larger size of older larvae, however, may draw more attention from predators and may offset the shorter time they are exposed near the water surface. Thus, both small and large larvae have some advantages and disadvantages in avoiding predation by *O. chalybescens*. An additional subtlety lies in their low capture rate of mosquito pupae, possibly because of relative immobility in this life stage (Minakawa *et al*., 2007).

Early studies found *Ochthera brevitibialis* preying on anopheline mosquito larvae and bloodworms. This species of shore fly was sometimes sufficiently numerous to reduce local populations of Anophelines, but had no effect over a larger area. The Anophelines were observed to be easier to catch than *Culex* spp. in deeper water as the latter were able to escape more readily (Travis, 1947).

The Chaoboridae, commonly known as phantom midges or glassworms, have aquatic larvae and feed largely on small insects including mosquito larvae and crustaceans such as *Daphnia* (Cladocera: Daphniidae). There is no evidence that this predator specializes on mosquitoes (Bay, 1974).

#### True bugs (aquatic Hemiptera)

Hemiptera are mostly herbivorous and use their straw‐like mouthparts to inject enzymes into plants. These digest plant material, allowing the insect to suck the liquefied food back through its mouthparts and into its digestive tract. A few species of Corixidae (water boatmen) are predatory. These are generalist predators in aquatic insect communities, have great plasticity in prey choice and can remain abundant in varied resource environments (Symondson *et al*., 2002). Several studies have proposed aquatic hemipterans as potential mosquito control agents (Darriet & Hougard, 1993; Ohba & Nakasuji, 2006; Sivagnaname, 2009; Saha *et al*., 2012).

Ohba & Nakasuji (2006) investigated feeding habits of *Nepoidea* (Belostomatidae, water bugs, and Nepidae, water scorpions). They collected dietary items in wetlands and obtained data from the published literature that showed a broad diet. These species are also effective predators of medically important pests, such as snails, and mosquito larvae (39.3% of diet was insect, of which a proportion was mosquito). Examinations of the DNA of gut contents of invertebrate and vertebrate taxa in Kenyan wetlands revealed Nepomorpha (true water bugs) to be greater consumers of mosquitoes of human importance than were amphibians (Ohba *et al*., 2010). The major diet items of *Lethocerus deyrollei* (Vuillefroy) (giant water bug) (Hemiptera: Belostomatidae) and *Laccotrephes japonensis* (Scott) (water scorpion) (Hemiptera: Nepidae) are tadpoles, but *L. japonensis* nymphs also feed on aquatic insects, including mosquito larvae. The dominant feeding strategy in this taxon is predaceous, but several species consume other foods, particularly algae and detritus (Hadicke *et al*., 2017).

Jansson & Scudder (1972) observed *Cymatia* sp. (water boatmen) (Hemiptera: Corixidae) to capture mosquito larvae. Hale (1922) kept several Australian species of Corixinae (water boatman) in aquaria for months, and during that time fed them only with larvae of *Culex quinquefasciatus* and *Scutomyia notoscripta* (Diptera: Culicidae) mosquitoes. Newly hatched Corixinae captured early‐instar larvae and increasingly larger individuals during successive stages of metamorphosis.

Saha *et al*. (2010) investigated the prey electivity and switching dynamics of predatory heteropteran water bugs *Anisops bouvieri* (Hemiptera: Notonectidae), *Diplonychus rusticus* (Hemiptera: Belostomatidae) and *Diplonychus annulatus* in the laboratory under various prey densities using mosquito and chironomid prey. *Anisops bouvieri* and *D. rusticus* consume mosquito larvae under many circumstances but can readily forage on other prey when mosquito densities are low.

In India, the giant water bug *Diplonychus indicus* is a voracious predator of mosquito larvae, tadpoles and juvenile fish, with a preference for both anopheline and culicine larvae. A single water bug may consume 300 larvae per day (Venkatesan *et al*., 1986).

Munga *et al*. (2006), studied the effects of predator and competitor presence on the oviposition rates of *An. gambiae* in the laboratory. Rainwater was either conditioned, or not, with a backswimmer or a tadpole; and mosquitoes laid fewer eggs in conditioned than in unconditioned rainwater. Intraspecific competition tests showed that more mosquito eggs were laid in containers with five conspecific larvae than in those with higher densities (40, 70 or 100 larvae). Warburg *et al*. (2011) also found that when offered deionized water and deionized water conditioned with *Notonecta maculata* (Hemiptera: Notonectidae), gravid *An. gambiae* females preferentially oviposited into the predator‐free option.

#### Dragonflies and damselflies (Odonata)

Adult dragonflies are conspicuous predators of mosquitoes and are sometimes termed ‘mosquito hawks’. Most studies, however, have focused on larval predation. In field surveys of predators in Kenya (Service, 1973, 1977), serological studies of the gut showed that none of the larval Odonata tested positive for mosquito prey in 1973 and only half of the species tested positive in 1977. *Ischnura senegalensis* (Odonata: Coenagrionidae) was the most common mosquito feeder, with 47% testing positive for *An. gambiae*.

Saha *et al*. (2012) evaluated the predation potential of larvae of the damselfly *Ceriagrion coromandelianum* (Odonata: Coenagrionidae) and the dragonfly *Brachydiplax chalybea* (Odonata: Libellulidae) under varied habitat conditions. Odonate larvae were exposed to different densities of *Cx. quinquefasciatus* larvae in small water‐filled containers and then large containers with vegetation that provided a semi‐field environment. The presence of vegetation reduced predation of mosquito larvae by both species, possibly because plants reduce the effective space available for prey–predator interaction and serve as a refuge that reduces prey vulnerability (Saha *et al*., 2012).

Studies of the foregut contents of field‐caught juvenile *Enallagma civile* (Odonata: Coenagrionidae) damselflies revealed they had fed predominantly on chironomid larvae. Corixid, cladoceran, ostracod and aquatic mite remains were also found in some specimens examined. However, no remains of mosquito larvae were detected in any specimens, although mosquito larvae were observed as continuously present in sample pond sites (Breene *et al*., 1990).

Although odonate larvae are widely considered to be voracious predators of mosquito larvae, this is not supported by the available literature. They are true generalist predators with a wide range of dietary choice (Corbet, 1980).

#### Shrimps and other Crustacea

The presence of the tadpole shrimp *Triops granarius* (Notostraca: Triopsidae) was coincident with low numbers of *An. gambiae* larvae in temporary pools around huts in a village in Somalia (Maffi, 1962), although it was not clear whether this reflected avoidance, predation or competition. Laboratory oviposition choice tests and behavioural observations indicated that the activity of the tadpole shrimp *Triops longicaudatus* near the water surface deterred gravid *Cx. quinquefasciatus* from ovipositing (Tietze & Mulla, 1991). Consequently, *Triops* spp. are considered both effective larval predators and mosquito oviposition deterrents (Fry *et al*., 1994).

Field surveys of *Anopheles*, floodwater *Aedes* and *Culex* breeding habitats have shown that natural copepod populations can substantially reduce, or even eliminate, mosquito production. Field trials in temporary pools, marshes and rice fields have demonstrated that the introduction of the right copepod species to the right habitat at the right time can eliminate *Anopheles* or floodwater *Aedes* larvae. Cyclopoid copepod predators consume protozoans, rotifers and small aquatic animals such as first and second instar mosquito larvae (Marten *et al*., 1994; Marten & Reid, 2007).

#### Spiders (Arachnida)

Spiders feeding in and around aquatic habitats have a diverse array of strategies; most spiders that predate on mosquito larvae are active hunters that do not build webs. These spiders can be terrestrial, standing at the water's edge, semi‐aquatic, surface film locomotors or subsurface divers that use air sacs.

Using the serological method, Service (1971, 1973, 1977) identified several predators of *An. gambiae* in western Kenya, among which, wolf spiders (Lycosidae) were important consumers of newly emerged adults. By contrast, Perevozkin *et al*. (2004) found that spiders belonging to the genera *Dolomedes* (Araneae: Pisauridae) and *Argyroneta* (Araneae: Cybaeidae) actively preyed upon anopheline and culicine larvae, but wolf spiders [*Pardosa* spp. (Araneae: Lycosidae)] did not. Perevozkin *et al*. (2004) then conducted experiments which showed that preying upon aquatic organisms, including malaria mosquito larvae, is not habitual for *Pirata* spp. (Araneae: Lycosidae) and *Pardosa* spp. spiders. These predators are terrestrial and find the bulk of their prey on land near the water's edge.

The semi‐aquatic spiders of the genus *Dolomedes* are surface film locomotors and active predators of mosquito larvae as part of a broad diet (Zimmermann & Spence, 1989). When fed only *Anopheles* larvae, they grew normally and successfully completed development. *Argyroneta aquatica* is another active predator of anopheline mosquitoes. These predate underwater using a network of threads surrounding a bell‐shaped nest made of silk and submerged aquatic plants. The spider fills the nest with air and hides inside while trapping its prey (Perevozkin *et al*., 2004). The food preferences of *Dolomedes and Argyroneta* were found to depend on the size of their prey and its mode of locomotion. *Argyroneta aquatica* preferred hunting *Anopheles* larvae, irrespective of differences in body size between them and *Culex* larvae. *Anopheles* larvae move jerkily in water and these movements attracted the spider; *Culex* larvae glide more smoothly.

Futami *et al*. (2008) also studied the predatory ability of wolf spiders alongside the predator avoidance techniques of *An. gambiae*. Diving ability develops with instar stages of *An. gambiae* (Tuno *et al*., 2007), which may affect predation by wolf spiders. Although predation intensity was low for first instars, separate experiments showed that spiders could capture nearly 50% of second, third and fourth instars within 24 h. General mortality increased with mosquito age, except for pupae. Fewer pupae were captured, possibly because they are less active; when they do move, they do so quickly and their smooth round shape makes them harder to grip. These results, along with all other laboratory tests, cannot be directly extrapolated to describe the predatory capacity of this spider in nature.

#### Flatworms (Planaria)

Planarians are free‐living flatworms and form the traditional class Turbellaria in the phylum Platyhelminthes*;* other classes of flatworms are parasitic. A rhabdocoele turbellarian is identified as predatory on aquatic arthropods, including aquatic stages of *An. gambiae s.l*. One worm can kill an individual larva, but the individual attack is typically followed by a mass attack (Mead, 1978). Kar & Aditya (2003) demonstrated how larvae of mosquitoes can be consumed by a species of planarian, *Dugesia bengalensis* (Tricladida: Dugesiidae). A laboratory experiment showed that planarians prefer the second and third larval stages of mosquitoes (*Anopheles* and *Culex*) in which the exoskeleton is not yet fully hardened. No alternative prey were offered and hence the extent of consumption or preference for mosquito larvae in the wild is not known. The most important flatworm predators are species of *Mesostoma* (Typhloplanoida: Typhloplanidae) that occur in a wide range of habitats. Single prey experiments show that a number of *Mesostoma* species feed heavily on mosquito larvae, some chironomid larvae and some daphnids, but considerably less on copepods and ostracods. Prey preference experiments reflect the same trends (Blaustein & Dumont, 1990).

#### Frogs, toads and tadpoles (Amphibia, Anura)

When frogs and toads consume mosquitoes, it is usually thought to be as adults. Tadpoles are often largely herbivorous, although some larger species do prey on mosquito larvae. That said, the majority of the published research is focused on tadpoles. The dietary niche breadth of larvivorous tadpoles includes other predators of mosquito larvae. Their consumption of mosquito larvae in the presence of alternate prey has not been properly elucidated (Kumar & Hwang, 2006).

Detailed studies by Service (1973) found both adult frogs and tadpoles in experimental ponds, small pools and ditches, but only tadpoles were found to consume *An. gambiae s.l*. [*Hyperolius* sp. (Anura: Hyperoliidae) and *Phrynobatrachus* sp. (Anura: Phrynobatrachidae)]. Service (1977), in a similar study, found both frogs and tadpoles but again only the tadpole gut content tested positive for *An. gambiae s.l*. [*Phrynobatrachus* sp. and *Ptychadena* sp. (Anura: Ptychadenidae)]. It is possible that the larger size and more rapid digestion rate of adult frogs does not lend itself to this method of testing stomach contents. The Asian bullfrog tadpole *Hoplobatrachus tigerinus* (Anura: Dicroglossidae) has been shown to effectively prey on larvae of *Aedes aegypti*; under laboratory conditions consumption per tadpole per day amounted to 29.0 ± 2.0 first instar larvae (Murugan *et al*., 2015).

#### Fish (Osteichthyes)

Fish predation of mosquito larvae has been recorded in many habitats, from experimental small plastic containers to complex natural ecosystems. Larvivorous fish have been demonstrated to be effective at reducing larval mosquito populations in many parts of the world, through direct predation, and inhibition of development and of oviposition.

Predatory and non‐predatory effects of the mosquito fish *Gambusia affinis* (Cyprinodontiformes: Poeciliidae) and *Carassius auratus* (Cypriniformes: Cyprinidae) on both gravid *An. gambiae s.s*. and larval survivorship revealed direct and indirect effects on life history traits of *An. gambiae s.s*. The presence of a predator reduced the number of larvae, but also reduced their growth and developmental rate, and hindered oviposition in potential larval habitats (Chobu *et al*., 2015). Similar findings have been reported in other studies conducted in both laboratory and semi‐field settings (Munga *et al*., 2006; Kweka *et al*., 2011). Physiological stress induced by the presence of predators can reduce adult body size and consequently their mating, fecundity, oviposition and survivorship potential.

The larvivorous fish *Oreochromis spilurus* (Cichliformes: Cichlidae) (a tilapia) was assessed as an agent of malaria vector control in northern Somalia. The mean reduction observed in the field was 52.8%. In laboratory studies, the fish consumed all available larvae, but this did not occur in the field, where other foods were available and habitats offer options for larval predator avoidance (Mohamed, 2003).

Louca *et al*. (2009) conducted semi‐field and field trials to look at comparative abundances of *Anopheles* and *Culex* mosquitoes in relation to native fish populations. Another semi‐field trial tested the predatory capacity of fish on mosquito larvae and influence of fish chemical cues on oviposition. In this, both *Tilapia guineensis* (Perciformes: Cichlidae) and *Epiplatys spilargyreius* (Cyprinodontiformes: Aplocheilidae) were effective predators, removing all late‐stage culicine and anopheline larvae within 1 day. In the field, there was less chance of finding culicine larvae where *T. guineensis* was present; however, the presence of Anophelines was not related to the presence or absence of any fish species. These studies indicate that although *T. guineensis* is a predator of mosquito larvae, it is not a specialist (Kumar & Hwang, 2006; Louca *et al*., 2009).

Kweka *et al*. (2011) found survival rates in semi‐field experiments varied with predator species, but not larval density and habitat type, reflecting the combined effects of searching and consumption of *An. gambiae s.l*. larvae by predator species in both time and space. Studies of the gut content of *Gambusia holbrooki* showed these fish to be true generalists and recorded 34% algae and 19% detritus, in addition to invertebrate prey. Animal consumption included 11% rotifers, 28% dipterans, 19% ostracods, 19% other insects, 18% copepods and 5% cladocerans (Garcia‐Berthou, 1999; Specziár, 2004). Hence, although potentially a contributor to biological control of mosquitoes, all fish, including the mosquito fish *G. affinis*, are flexible and generalist predators.

Although mosquito larvae are often proposed as important in the natural diet of fish, exclusions of mosquitoes from aquatic habitats caused by predation, or avoidance by ovipositing female mosquitoes, have rarely been studied. In Colombia, larvae of *Anopheles albimanus* were negatively associated with fish and predatory invertebrates, such as dragonfly and mayfly nymphs. These fish maintain their niche occupation whether or not mosquito larvae are present (Marten *et al*., 1996).

#### Birds (Aves)

Many of the birds that make use of freshwater habitats are insectivorous and are likely to feed on mosquito larvae as part of a broad opportunistic diet. There is little quantitative evidence of specific mosquito consumption in the aquatic larval habitat. Further information on predation by birds is given under the section on predation of adults.

#### Parasite species of aquatic habitats

Studies by Service (1973, 1977) revealed that a high proportion (82–88%) of fourth instar larvae collected from ponds were infected with larval nematodes, whereas only 8–15% of larvae in ditches had nematodes, and no larvae in transient pools did so. Most infected larvae (51–57%) had one larval nematode in their body cavity, but a few (2.9%) had as many as five nematodes.

Overall, 77% of third and fourth instar larvae from ditches and 95% from pools were infected by *Coelomomycete* fungi (Blastocladiales: Coelomomycetaceae), but not larvae from ponds. No pupae were found in the ditches and only five pupae were found in other habitats where *Coelomomyces* was present. A number of infected larvae taken to the laboratory pupated, but no adults emerged and all pupae were found to be infected with *Coelomomyces*. Similar percentages were reported in Service (1977). Infections with *Coelomomyces* usually result in mortality before pupation and females that emerge from infected immature stages do not produce eggs (Service, 1973, 1977).

### Predation of adults

In *Anopheles* mosquitoes, wild adult males are estimated to live for less than 2 weeks, feeding on nectar and other sources of sugar. Females also feed on sugar sources but require a bloodmeal to develop eggs. Most females probably do not live longer than 1–2 weeks in nature. Their chances of survival depend on temperature and humidity, but also on their ability to successfully obtain a bloodmeal while avoiding host defences and predation.

*Anopheles gambiae s.l*. is highly anthropophilic (preferring human beings to other animals), although its host selection is influenced by location, host availability and the genetic make‐up of the mosquito population. Female *An. gambiae* typically feed late at night and are often described as both endophagic and endophilic (feeding on humans indoors as they sleep). Yet there is evidence that indoor and outdoor biting are common and both indoor and outdoor resting behaviours are reported (Silver, 2008). As with host preference, this species appears to exhibit phenotypic plasticity (adaptation to different conditions) and opportunism in resting locations.

#### Flies (Diptera)

Adult shore flies *O. chalybescens* have been observed to prey on emerging adult anopheline mosquitoes in western Kenya. Many insects are most vulnerable to predation during emergence from the pupal stage, when their bodies have not yet fully hardened (Minakawa *et al*., 2007).

#### Dragonflies and damselflies (Odonata)

There is little quantitative information on adult dragonflies as predators. They feed on ants, termites, butterflies, gnats, bees and flies. As male *Anopheles* mosquitoes aggregate in mating swarms at dusk, researchers have observed dragonfly predation (C.M. Collins & J.A.S. Bonds; personal observation, 2017). These swarms are very short‐lived and unlikely to contribute significantly to dragonfly diet. Yuval & Bouskila (1993) determined numbers of copulations and predatory attacks in mating swarms of *Anopheles freeborni* over 19 evenings of observation and recorded 2724 copulating pairs leaving swarms and 1351 dragonfly [*Pantala hymenaea* and *Erythemis collocata* (both: Odonata: Libellulidae)] attacks.

#### Spiders (Arachnida)

Wesolowska & Jackson (2003) presented field data on the natural diet of *Evarcha culicivora* (Araneae: Salticidae), a salticid jumping spider from the shores of Lake Victoria in East Africa, showing that adult female mosquitoes are the dominant prey. Adult female mosquitoes, independent of a spider's sex or age class, constituted around 70% of prey caught. Of those identified, just under half were *An. gambiae s.l*. and just over half were *Culex* spp. The second most important prey group were non‐mosquito adult Diptera. Insects other than dipterans accounted for only 3%.

In laboratory preference studies, Pollard & Jackson (2007) investigated predation rates by *E. culicivora* on mosquitoes and lake flies. Lake flies resemble mosquitoes in general body form and size, and vastly outnumber mosquitoes, but are not blood feeders. These studies suggest that *E. culicivora* expresses a preference for mosquitoes over non‐mosquito prey, for blood‐carrying instead of non‐blood‐carrying mosquitoes and for *Anopheles* over other mosquito genera (Jackson *et al*., 2005; Nelson & Jackson, 2006).

Predator size affects this preference. Nelson & Jackson (2006) showed that *E. culicivora* can identify non‐moving *Anopheles*. When the alternative prey offered is a blood‐fed *Culex*, small spiders maintain a preference for blood‐fed *Anopheles*, but there was no evidence that large, fasted spiders discriminated between blood‐carrying *Anopheles* and *Culex* (Nelson & Jackson, 2006). Later studies showed that the preference of *E. culicivora* for blood‐fed female anopheline mosquitoes was unique and not widespread in East African salticids. When live prey choice tests were carried out in 19 additional species, there were no instances in which blood‐carrying mosquitoes were chosen significantly more often than other prey (Jackson & Nelson, 2012).

After almost two decades of research and on the basis of 1115 records of *E. culicivora* feeding in the field, this East African jumping spider is considered stenophagic. Field prey belonged to 10 arthropod orders, but 94.5% were dipterans. Mosquitoes dominated (80.2% of the records), with the majority of mosquitoes (82.9%) being female, and hence 63.0% of the diet of these spiders consists of female mosquitoes. These authors make a clear distinction between preference and natural diet that is important when discussing predatory specialization because, although natural diet is simply what a predator eats in the field, preference is an inherent product of a predator's perceptual processes, decision‐making capacities and motivation. In terms of dependence, there is no evidence that these Salticids require *Anopheles* mosquitoes and they will readily feed on blood‐fed *Culex* (Jackson & Cross, 2015; Jackson *et al*., 2016).

#### Bats (Mammalia, Primata)

Insectivorous bats are often considered an important biological control for mosquito populations. Mosquitoes, however, represent only a small proportion of bat diet. Larger bats tend to use low‐frequency echolocation to detect higher‐value, larger prey, and the longer wavelength of this echolocation is unsuitable for detecting small prey such as mosquitoes (Barclay & Brigham, 1991).

No specific studies on *An. gambiae* were found, although Gonsalves *et al*. (2013) investigated whether consumption of mosquitoes was influenced by bat size. They studied diets of five eastern Australian bat species ranging in size from 4 g to 14 g. Using molecular analysis of faecal DNA, a diverse range of prey was detected. Lepidoptera (moths) dominated and analyses reflected their relative abundance at trap sites. Consumption of mosquitoes was restricted to two smaller bat species (4 g and 4.5 g). Although mosquitoes were not commonly detected in the faeces of one, *Vespadelus pumilus* (Chiroptera: Vespertilionidae), they were present in the faeces of 55% of *Vespadelus vulturnus* individuals. To meet nightly field metabolic rate requirements, *Vespadelus* spp. would need to consume ∼ 600–660 mosquitoes on a mosquito‐only diet, or ∼ 160–180 similarly sized moths on a moth‐only diet. The lower relative profitability of mosquitoes may provide an explanation for the low level of mosquito consumption among these bats and the absence of mosquitoes in faeces of larger bats. In Swaziland, a similar study of the diets of two sympatric free‐tailed bats, *Chaerephon pumilus* and *Mops condylurus* (both: Chiroptera: Molossidae), concluded these bats to be polyphagous, and found that although mosquitoes represented part of their diets, lepidopterans made up the majority (Bohmann *et al*., 2011).

#### Birds (Aves)

The most common mosquito‐eating birds are swallows, martins, warblers, waterfowls and sparrows. Insectivorous birds do not just consume adult mosquitoes: waterfowl in particular will also consume mosquito larvae, but there are no recorded mosquito‐specialist predators and birds generally have diverse diets (Batzer & Wissinger, 1996).

Without focusing on *Anopheles* spp., Poulin *et al*. (2010) used the house martin, *Delichon urbicum* (Passeriformes: Hirundinidae), as a model species to assess the ecosystem effects of a broad‐scale reduction of Nematocera (a Dipteran suborder that includes midges and mosquitoes) in Camargue, France by biological control using *Bacillus thuringiensis* subspecies *israelensis* (*Bti*), a naturally occurring soil bacterium used in biological control of some Diptera, including mosquitoes. Clutch size and fledgling survival were lower at treated sites relative to control sites (2.3 vs. 3.2 chicks produced per nest). Intake of Nematocera and their predators (spiders and dragonflies) decreased at treated sites, and flying ant consumption increased. Later studies showed that this broad‐spectrum treatment also reduced populations of Odonata (Jakob & Poulin, 2016). Timmermann & Becker (2017) also investigated direct and indirect effects of large‐scale *Bti* treatment on food sources for *D. urbicum* in the Upper Rhine Valley and concluded that not even the direct effect of reduction of the mosquito population made a difference in diet abundance because the overall percentage of the total insect biomass represented by mosquitoes was low.

Other studies indicate that, much like bats, birds tend toward larger, more rewarding prey. For example, the diet of the house wren, *Troglodytes aedon* (Passeriformes: Troglodytidae), was determined from the gut contents of wrens collected on a forested dune ridge at Delta Marsh in Manitoba, Canada. Prey selection depended on abundance, size and ease of capture (Guinan & Sealy, 1987). According to ornithologist James Hill, ‘The number of mosquitoes that martins eat is insignificant. In‐depth studies have shown that mosquitoes comprise no more than 0 to 3 percent of the diet of martins’ (Hill, 2017). In another study of diets of sand martins, Culicines were identified to be present in only 0.33% of faecal samples (Waugh, 1979).

### Interactions with competitor species

Intra‐ and interspecific competition only occur when a resource, such as space, shelter, food, micronutrients and mates, is in short supply. There are many studies of competition both within and between *Anopheles* species in larval habitats, although competition, other than for mating opportunity, is largely unexplored during the adult life stage. A significant issue in many larval studies is the balance of tractability to realism: a high proportion of these studies are performed in simplified micro‐ or mesocosms and thus in environmental conditions that are less variable than conditions in the field; this can limit the realism of their extrapolation.

Intraspecific competition can constrain the local abundance of a species, but interspecific competition can constrain the abundances of other species co‐occurring in the habitat. The term ‘competitive displacement’ is used when one species with any competitive advantage over another is, in a stable environment, able to dominate that habitat or ecological niche. In other words, that species' strength as a competitor restricts or displaces other species. If a species is reduced in numbers over time and space, then other species that have previously been displaced may increase in number; this is termed ‘competitive release’. The concept in terms of mosquito control is described in detail by Lounibos (2007).

#### Inferences from mosquito control interventions

Many mosquito control programmes have been successful and provide evidence of the effects of reduced competition from a focal species. In early 20th century Italian mosquito control, the salt‐tolerant vector *Anopheles labranchiae* was largely replaced by the zoophilic *Anopheles hispaniola* following the desalination of larval habitats (Missiroli, 1939). This shift in balance between two anopheline species in southern Italy was not permanent; over the following 35 years, the number of sites occupied by *An. labranchiae* rose and *An. hispaniola* disappeared from several areas, including Sardinia (Marchi & Munstermann, 1987).

In the 1950s, IRS with dieldrin to control malaria in southern Kenya and northern Tanzania led to the virtual disappearance of the vector *Anopheles funestus* and its replacement by the related, but zoophilic, species *Anopheles rivulorum* (Gillies & Smith, 1960). As larvae of the two species frequently co‐occur, these authors conjectured that interspecific larval competition had suppressed *An. rivulorum* numbers prior to the high mortality suffered by *An. funestus* from insecticide treatments. The decrease in *An. funestus* abundance released *An. rivulorum* from competition and the niche dominance of the former species in their shared habitats. A later study (Gillies & Furlong, 1964) also reported the reduction of *An. funestus* after an IRS project on the Kenyan coast. The sibling species *Anopheles parensis*, an exophilic mosquito, increased in both relative abundance and absolute numbers, possibly again as a result of reduced competition in larval habitats.

A marked decline in the *An. gambiae s.s*. population in response to an ITN programme was similarly noted in a region of western Kenya (Bayoh *et al*., 2010). Increased ITN coverage led to a reduction in the number of *An. gambiae s.s*. females ovipositing. *Anopheles gambiae s.s*. and *An. arabiensis* share the same larval habitats, but typically *An. gambiae* is more abundant. Its reduction may have released *An. arabiensis* from an aspect of larval competition and thus contributed to an increase in the latter's relative density. The trial showed that indoor density of *An*. *gambiae* diminished substantially and was followed by a reduction in malaria transmission as *An. arabiensis* is considered a less effective malaria vector with a preference for cattle. Thus, competitive release may have occurred, but led to a shift from anthropophilic to zoophilic mosquitoes.

A combination of mosquito control and land use change provides an interesting example of the effects of altered competition on the ecology of a species. After the elimination of *Anopheles darlingi* and malaria in the Demerara River Estuary of Guiana (by DDT spraying), the human population grew rapidly and land use activities switched from livestock to more profitable rice farming. The removal of livestock from the landscape, however, caused the formerly zoophilic *Anopheles aquasalis* to switch its feeding from livestock to humans. This change initiated the return of transmission to the area after 16 years of absence (Giglioli, 1951, 1963).

#### Interspecific competition within the species complex: An. gambiae s.l.

Many studies highlight temporal and spatial variations in abundances of different species within the *An. gambiae* complex. Most of these studies focus on direct competition between the three principal malaria vectors: *An. gambiae*, *An. coluzzii* and *An. arabiensis*. Although largely sympatric with larvae found occupying the same habitats (e.g. man‐made holes, roadside ditches, transient puddles and footprints), *An. arabiensis* is considered to be better adapted to dry, hot conditions, whereas *An. gambiae s.s*. is the superior competitor in wetter field conditions (Githeko *et al*., 1996; Schneider *et al*., 2000; Gimnig *et al*., 2001; Koenraadt *et al*., 2004b). Differences in the survival and development of aquatic larval stages of these species at different temperatures may help to explain adult distributions in much of Africa (Lindsay *et al*., 1998; Coetzee *et al*., 2000).

In the absence of predation, the typically small breeding sites of *An. gambiae* can support very high larval densities, thus promoting the importance of intra‐ and interspecific competition. Mixed‐species rearing has been shown to have a detrimental effect on the survival of *An. arabiensis*, but not that of *An. gambiae*, at 30 °C (Gimnig *et al*., 2001, 2002). Schneider *et al*. (2000) found similar evidence and suggested that the larger *An. arabiensis* may have greater food requirements than *An. gambiae*. Thus, a limited food resource might impose relatively more competition on *An. arabiensis* than on *An. gambiae*. In further detailed *in vitro* experiments, larval development time was not affected by the presence of the other species. *Anopheles arabiensis* adults from both single‐ and mixed‐species containers developed more slowly and were consistently larger than those of *An. gambiae* at all temperatures. Adult size and the ability to survive desiccation and higher temperatures influence the outcome of competition in larval habitats and the population size achieved in time and space (Kirby & Lindsay, 2004, 2009).

More recently, laboratory and semi‐field trials have explored interspecific competition between *An. gambiae* and *An. coluzzii* larvae. Semi‐field study results using wild mosquitoes indicated that *An. gambiae* larvae are superior competitors to *An. coluzzii*. The reverse was true in insectary‐sourced and laboratory‐conducted trials, which suggests that the results of such laboratory studies of competition should be extrapolated only with caution to field populations (Gimonneau *et al*., 2014). Faster development of *An. gambiae* than of *An. coluzzii* in the field has also been reported in Burkina Faso (Diabaté *et al*., 2008). Indirect competition between *An. gambiae* and *An. coluzzii*, mediated via either predation or resource constraint, is of increasing interest and is likely to contribute to segregation of larval habitat (Gimonneau *et al*., 2012).

The competitive ability of *An. gambiae* has implications for species distribution and aquatic habitat colonization. Faster development time in temporary aquatic habitats is a plastic phenotypic trait of major importance that enables a species to reduce its exposure to negative effects such as desiccation (Lee *et al*., 2009; Benedict *et al*., 2010), predation risk (Gimonneau *et al*., 2010), cannibalism (Koenraadt & Takken, 2003; Muturi *et al*., 2010), pathogens (Ward & Savage, 1972) and flushing caused by rainfall (Paaijmans *et al*., 2007).

#### Interspecific competition: other dipteran genera

Interspecific competition with other genera of co‐occurring Diptera could lead, in some cases, to population‐level effects. Kweka *et al*. (2012) found effects on adult size, sex ratio and speed of development in *An. gambiae s.s*. in the presence of the mosquito *Cx. quinquefasciatus* (cohabitation treatment) in a semi‐field situation. The sex ratios of both *An. gambiae s.s*. and *Cx. quinquefasciatus* were male‐biased in single‐species treatments, whereas in cohabitation treatments the reverse was true. For both species, more pupae and adults emerged earlier from single‐species treatments than in cohabitation treatments. Increased larval density in both single‐ and mixed‐species treatments did not affect survival. Wing length, a proxy for overall body size, host‐seeking ability and fecundity in female mosquitoes, was reduced in *An. gambiae s.s*. by this interspecific competition. In other studies, laboratory experiments have shown that smaller female malaria vectors feed more frequently and may need two feeds before their first oviposition, and suggest this may influence arbovirus transmission. In cohabitation treatments niche partitioning was seen; *Cx. quinquefasciatus* fed on lower surface micro layers, whereas *An. gambiae s.s*. fed on upper surface micro layers. The authors suggest neither can lead to exclusion of the other in the conditions tested (Kweka *et al*., 2012).

## Conclusions

*Anopheles gambiae* is a species of importance because of its role as a vector of malaria, not as a key component of ecosystem food webs. The present comprehensive exploration of the literature, summarized in Tables 1 and 2, confirms the predictions of foraging theory. Adult *An. gambiae* mosquitoes are a relatively low‐value, low‐volume and disaggregated resource and this is reflected in a lack of evidence for any tight links with predators. No predators are recorded as being closely associated or dependent on larvae of these mosquitoes. The high seasonality of *An. gambiae* throughout most of its range and the ephemeral nature of many of its larval habitats also limit predation to generalist species that may take it as prey when the opportunity occurs. This generalist predation is a known stable strategy in ecological theory and contributes to dynamic equilibria in predator and prey populations and in the ecosystem in general.

**Table 1:** Overview of evidence of invertebrate predator interactions with larval and adult *Anopheles gambiae s.l*.

Predator group	Larva	Adult
**Flies (Diptera)**	Many species of generalist predatory dipteran larvae have been recorded in aquatic habitats. There is no evidence of specialism on *An. gambiae s.l*. in any.	Shore flies (*Ephydridae*) have been observed predating adult mosquitoes, but these curious flies do not specialize on *An. gambiae s.l*.; they are opportunistic generalist predators.
**True bugs (Hemiptera)**	There is no literature which suggests that any hemipteran predators specialize on *An. gambiae s.l*.; they are broad, generalist predators. There is some evidence that female mosquitoes will avoid oviposition in water that contains hemipteran predators.	No evidence of interaction.
**Dragonflies and damselflies (Odonata)**	Odonata are considered to be voracious predators of mosquito larvae. This, however, is not supported by the available literature. The odonate larvae are true generalist predators, with a wide range of dietary choice.	Several species of Odonata are predators of adult mosquitoes and have been seen to feed on male swarm aggregations, but there is no evidence indicating that they are specialist predators of *An. gambiae s.l*.
**Shrimps and others (Crustacea)**	Crustacean predators are widely present in more established water bodies. With a broad diet, these are classified as generalist predators. Their presence can also deter mosquito oviposition.	Predation not present outside the aquatic environment.
**Spiders (Arachnida)**	Many of the studies identified do provide some basic information on predation by aquatic or peri‐aquatic hunting spiders, but there is no evidence of diet specialization on mosquito larvae and these are considered generalist predators.	A single species of jumping spider (Salticidae) has been found to predate preferentially on blood‐fed female mosquitoes when they are resting to digest their bloodmeal. Female mosquitoes make up 63% of the *Evarcha culicivora* diet. This predatory spider with a relatively narrow diet range has a restricted distribution in East Africa near the shores of Lake Victoria.
**Flatworms (Planaria)**	Although some planarians will readily consume mosquito larvae, these are generalist predators.	Predation not present outside the aquatic environment.

**Table 2:** Overview of evidence for vertebrate predator, parasitic species and competitive interactions with larval and adult *Anopheles gambiae s.l*.

Vertebrate predators	Larval	Adult
**Fish (Osteichthyes)**	Insectivorous bony fish are naturally present in many stable longterm aquatic habitats. Mosquito larval density varies with fish presence, but fish presence has not been shown to vary with *Anopheles* larval presence. Insectivorous fish have a diverse diet and even those proposed as biological control agents of mosquitoes are not specialists of *An. gambiae s.l*.	Predation not present outside the aquatic environment.
**Bats (Mammalia, Primata)**	Predation not present in the aquatic environment.	The few detailed studies of bat diet available indicate clearly that mosquitoes form a small proportion of bat diet.
**Birds (Aves)**	Many birds that make use of freshwater habitats are insectivorous and thus likely to feed on mosquito larvae as part of an opportunistic broader diet. There is little quantitative evidence of specific mosquito consumption in the aquatic larval habitat.	Insectivorous birds are generalist predators; *An. gambiae s.l*. mosquitoes are not a significant portion of their diet.
**Parasitic species in aquatic habitats**		
It is likely that there are numerous parasites of mosquitoes; the most documented are fungi and nematodes. Although evidence points to high infection rates in many natural habitats, no evidence suggests any specialization on *An. gambiae s.l*.		
**Interactions with competitor species in aquatic habitats**		
There is some evidence from laboratory studies and previous mosquito control interventions that other mosquito co‐occupants of the larval habitat respond positively to a decrease in *Anopheles* species and specifically to reduced *An. gambiae s.s*. density. This is thought to result from reduced competition within the aquatic habitat that may lead to the competitive release of weaker competitor species.		

Several competing mosquito species could increase if *An. gambiae* density is reduced in specific habitats. Many generalist predators of *An. gambiae* already prey on these species and would substitute them for *An. gambiae* if the latter were less abundant. In this sense, any positive effects of competitive release on abundances of other mosquito species have the potential to compensate for any reduction of *An. gambiae* biomass in a diet. In terms of competing species that also act as vectors, *An. gambiae* is the most efficient vector of malaria (Lindsay *et al*., 1998) and malaria is a more significant cause of human mortality than diseases transmitted by other mosquito species.
